# Prognostic Pathways Guide Drug Indications in Pan-Cancers

**DOI:** 10.3389/fonc.2022.849552

**Published:** 2022-03-14

**Authors:** Fanlin Meng, Kenan Zhang, Changlin Yang, Ke Zhang, Quan Xu, Ruifang Ren, Yiming Zhou, Yimin Sun, Yan Peng, Yanze Li, Hongyan Guo, Yonghong Ren, Zheng Zhao

**Affiliations:** ^1^ Marketing and Management Department, CapitalBio Technology, Beijing, China; ^2^ National Engineering Research Center for Beijing Biochip Technology, Beijing, China; ^3^ Beijing Neurosurgical Institute, Capital Medical University, Beijing, China

**Keywords:** pathways, pan-cancers, “TCM-pathways-cancers” triplets, traditional Chinese medicines (TCMs), herbs

## Abstract

Pathway-level analysis is a powerful approach enabling the interpretation of post-genomic data at a higher level than that of individual molecules. Molecular-targeted therapy focusing on cascade signaling pathways has become a new paradigm in anticancer therapy, instead of a single protein. However, the approaches to narrowing down the long list of biological pathways are limited. Here, we proposed a strategy for *in silico* Drug Prescription on biological pathways across pan-Cancers (CDP), by connecting drugs to candidate pathways. Applying on a list of 120 traditional Chinese medicines (TCM), we especially identified the “TCM–pathways–cancers” triplet and constructed it into a heterogeneous network across pan-cancers. Applying them into TCMs, the computational prescribing methods deepened the understanding of the efficacy of TCM at the molecular level. Further applying them into Western medicines, CDP could promote drug reposition avoiding time-consuming developments of new drugs.

## Introduction

Advances in RNA sequencing technologies provide unprecedented capacity to comprehensively identify altered genes and pathways implicated in tumorigenic processes, raising the hope of extending targeted therapies (1–3). Monitoring the transcriptome of tumor samples mirrors voluminous altered pathways during cell programming and even heterogeneous cancer initiation and progression (4–6). Pathway-level transcriptomic analysis is a powerful approach to interpreting post-genomic data at a higher level than that of individual biomolecules (7, 8).

Tumor innate or acquired resistance leads to treatment failure (9). However, the development of new cancer therapeutics currently requires a long and protracted process of experimentation and testing (10). Recent research showed that the proportion of cancer patients who could be tractable by approved agents following clinical indications is merely 5.9% in a cohort of 4,068 tumors (11). Most of the single-agent drugs under development today target a specific protein (12). However, the formation and progression of a tumor usually occur through a range of defects in endothelial cells, immune cells, stromal cells, and cancerous cells (13). Defects in the whole signaling pathways allow cancer cells to alter their normal programmers of proliferation, transcription, growth, migration, differentiation, and death (14). Therefore, targeting multiple molecules, i.e., cascades of signaling pathways involved in both tumor and surrounding tissues, appears to be a new paradigm for anticancer therapy, rather than a single protein (15, 16).

The high failure rate of chemotherapy and the tread toward multitarget paradigms emphasize the importance of discovering novel effective therapies. Discovered pathways have been proposed to accelerate the development and approval of effective and targeting cancer therapies, such as the PI3K/AKT/mTOR pathway in bladder cancer (17). Apoptosis pathway (18), Src kinase pathway (19), Hippo signaling pathway (20), Wnt pathway (21), and DNA repair pathway (22) were all proposed as therapeutical targets in cancers. Besides in cancer stem cells, Ivy’s group reviewed that targeting Notch, Hedgehog, and Wnt pathway to control stem-cell replication, survival, and differentiation is a new strategy under development (23). As emerging anticancer drug targets, all these pathways could make contributions to the development of the clinical treatments for cancers.

Here, we developed a strategy for *in silico* **D**rug (including herbal medicines) **P**rescription on cellular pathways in **C**ancers (CDP). Our CDP connected drugs (agents or perturbations or small molecules) to pan-cancers on a pathway level to discover novel effective therapies. First, CDP established a pathway dysregulation profile and identified pathway modes of action. Second, CDP determined the magnitude of pathways across 20 cancers based on a generality score, and the tumor specificity of pathways based on prognostic evidence. Third, we linked pathways with 1,309 drugs and connected cancers to all potential beneficial agents. We proposed two applications of CDP in drug design. First is the reposition of well-known drugs for new indications, which can not only save time and cost in the process of research and development but also avoid the side effects. Second is the combination of traditional Chinese medicines (herbal components) to prescribe new potential formula targeting cellular pathways.

## Results

### Pan-Cancer Pathway Panels Reveal Tumor Connections

**Figure 1:**
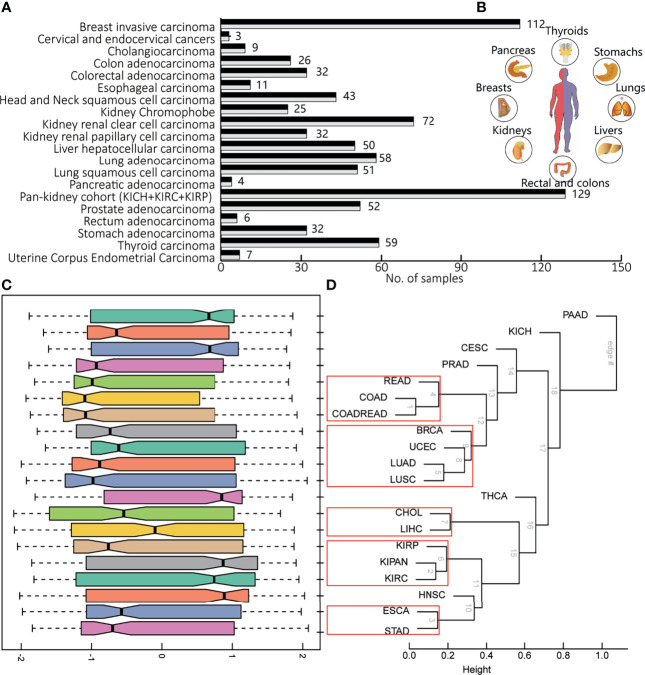
The brief of data source in this study. **(A)** The number of samples in the pan-cancer cohorts, one case–control study for each cancer type. **(B)** The tissues involved in pan-cancer, including livers, lungs, stomachs, thyroids, pancreas, breasts, kidneys, and rectal and colons. **(C)** The distribution pathway dysregulation reflecting by normalized enrichment scores (NES) in the form of boxplot. **(D)** Hierarchical clustering of various according to gene expression profiles of all detected genes, using the correlation and average linkage.

To portray the molecular pathway-level characterization of pan-cancer, we collected and integrated case–control studies of gene expression profiles and clinical outcome data from the Cancer Genome Atlas (TCGA) including 20 cancers. The paired pan-cancer cohorts contained 1,626 samples covering more than 10 distinct malignant histologies (**Figure 1A**, inlet). One tumor type corresponded to one case–control study, consisting of 6 to 224 normal and tumor samples (**Figure 1B**).

We constructed an activity profile of pathways based on those gene expression profiles of case–control studies across 20 cancers. The activity profile consists of normalized enrichment scores (NESs) for 178 pathways, representing the dysregulation (enrichment or depletion) score of each pathway in each tumor type. The **c**ancer-related **N**E**S** matrix was referred to as CNS.

The molecular characterization of pan-cancer at the pathway level revealed heterogeneity among different tumor tissues and consistency between tumor and supportive tissues. Hierarchical averaged-linkage clustering of 20 tumors showed 5 subcategories, with proximal to relevant tissues and organs or with similar physical functions: 1) READ, COAD, and COADREAD; 2) breast invasive carcinoma (BRCA), UCEC, LUAD, and LUSC; 3) cholangiocarcinoma (CHOL) and liver hepatocellular carcinoma (LIHC); 4) KIRP, KIPAN, and KIRC; and 5) esophageal carcinoma (ESCA) and stomach adenocarcinoma (STAD) (**Figures 1C, D**). PAAD, PRAD, and THCA seem to be quite different from other tumors across the deregulated features of KEGG pathways. Although these three cancers did not converge in one branch, evidence supported their underlying links (24). For most cancers, the corresponding distribution of NES indicated the overall level of pathway dysregulation within each subcategory (**Figure 1C**), consistent with the hierarchy shown in **Figure 1D**. For example, CHOL and LIHC are both hepatobiliary tumors. STAD and ESCA are both gastroenterological tumors. The abbreviations of all above cancers are shown in **Table 1**. Therefore, pathway panels in pan-cancers hinted the potential links underlying various tumors, suggesting subcategories among cancers.

**Table 1:** The numbers of samples in each cancer type.

TCGA label	Definition	N	N_tumor_	N_normal_
BRCA	Breast invasive carcinoma	224	112	112
CESC	Cervical squamous cell carcinoma and endocervical adenocarcinoma	6	3	3
CHOL	Cholangiocarcinoma	18	9	9
COAD	Colon adenocarcinoma	52	26	26
COREAD	Colon adenocarcinoma and rectum adenocarcinoma	64	32	32
ESCA	Esophageal carcinoma	22	11	11
HNSC	Head and neck squamous cell carcinoma	86	43	43
KICH	Kidney chromophobe	50	25	25
KIPAN	Pan-kidney cohort (KICH+KIRC+KIRP)	258	129	129
KIRP	Kidney renal papillary cell carcinoma	64	32	32
KIRC	Kidney renal clear cell carcinoma	144	72	72
LIHC	Liver hepatocellular carcinoma	100	50	50
LUAD	Lung adenocarcinoma	116	58	58
LUSC	Lung squamous cell carcinoma	102	51	51
PAAD	Pancreatic adenocarcinoma	8	4	4
PRAD	Prostate adenocarcinoma	104	52	52
READ	Rectum adenocarcinoma	12	6	6
STAD	Stomach adenocarcinoma	64	32	32
THCA	Thyroid carcinoma	118	59	59
UCEC	Uterine corpus endometrial carcinoma	14	7	7

### Pan-Cancer Pathway Panels Reveal Mode of Action (MoA) of Pathways

**Figure 2:**
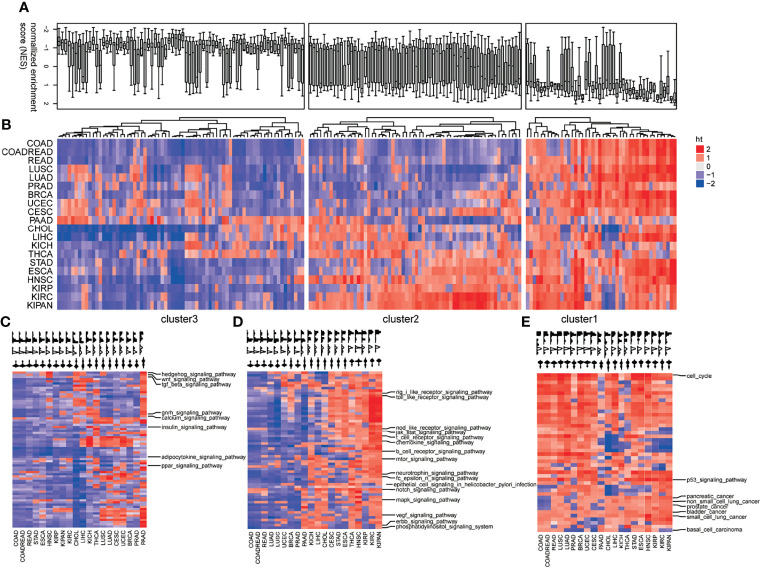
Profiles of pathway dysregulations on expression across pan-cancers. **(A)** The distribution pathway dysregulation reflecting by normalized enrichment scores (NES) in the form of boxplot. **(B)** The distribution pathway dysregulation reflecting by normalized enrichment scores (NES) in the form of heatmap. Three modes of action (MoA) of pathways: loss of functions (LoF) **(C)**, switch of functions (SoF) **(D)**, and gain of functions (GoF) **(E)**. Hierarchical clustering activity profiles (NESs) of 178 biological pathways based on GSEA, using the Euclidean distance metric and average linkage.

To investigate the pan-cancer pathway dysregulations across pan-cancers, the 178 KEGG pathways were partitioned into three clusters based on the CNS matrix (**Figures 2A, B**): loss of functions (LoF, Cluster 1), especially pathways harboring tumor-suppressive genes or downregulated genes in tumors, whose disruption facilitates tumorigenesis (**Figure 2C**, **Figure S1**); the LoF cluster which consists of 38 pathways including Hedgehog signaling, TGF-β, and WNT signaling; and switch of functions (SoF, Cluster 2) which defines the pathways whose favorable or adverse roles switched across different tumors (**Figure 2D**, **Figure S2**). A set of 62 cellular pathways were categorized into the SoF cluster, in which cellular signaling pathways accounted for a quarter. The well-known NOTCH, MAPK, mTOR, VEGF, JAK/STAT, and ERBB were grouped into the SoF cluster, indicating changes in their roles across various tumors. In addition, immune processes including B/T cell receptor signaling, chemokine signaling, and epithelial cell signaling in infection also play different roles in the tumor initiation and progression across various cancers (25). Gain of functions (GoF, Cluster 3) defines typically pathways consisting of oncogenic genes or unregulated genes in tumors, whose abnormal activation initiates tumorigenesis (**Figure 2E**, **Figure S3**). There were totally 72 cellular pathways categorized into the GoF cluster, including cell cycle, p53 signaling pathway, and basal cell carcinoma. **Figures 2A, B** shows the distribution of the dysregulations in each mode of action (MoA) in a boxplot and heatmap.

**Figure 3:**
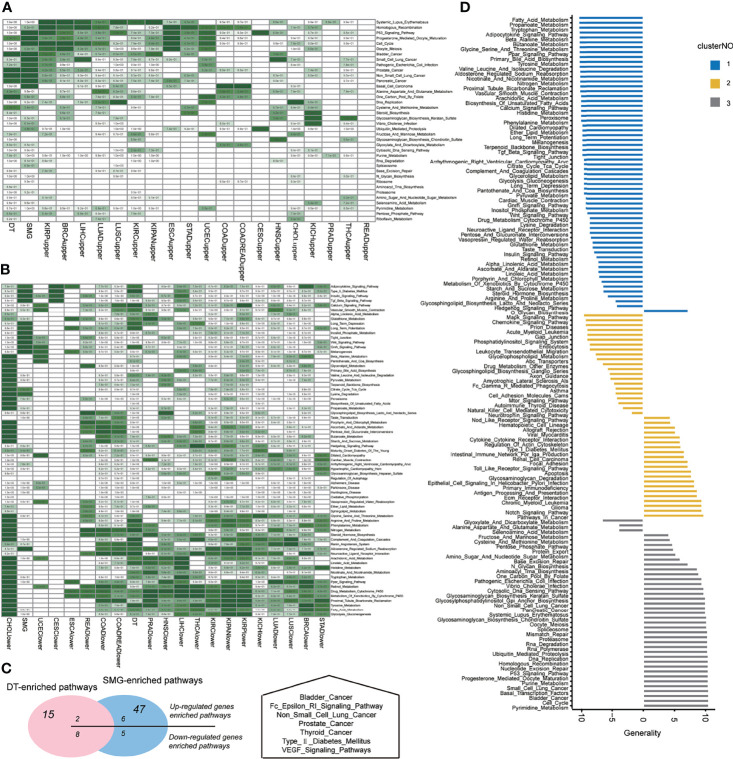
Pathway dysregulations on functions across pan-cancers. **(A)** The enrichment on the set of somatic mutations genes (SMGs) of differential expression genes in each cancer type. **(B)** The enrichment on the set of drug targets (DTs) of differential expression genes in each cancer type. Hypergeometric tests were performed to obtain statistical significance, i.e., *p* value. The blank cells in the heatmap means that the *p* value was 1. The darker the green color, the smaller the significant *p* value of the hypergeometric test. **(C)** The overlap of SMG-enriched or DT-enriched pathways on upregulated or downregulated pathways across pan-cancers, which indicated the potential of a pathway to be a target based on its mutation burden or target enrichment. **(D)** The distribution of generality scores across three clusters of pathways according to three MoAs.

To validate the oncogenic and tumor-suppressive characteristics of GoF and LoF pathways, we detected the differential expressed genes (DEG) in comparison of tumor and normal samples for each cancer type. Considering the robustness and the number of DEGs, we determined the optimal threshold (| log_2_(fold change) | > 2, FDR <0.01) for the differentially expressed analysis. **Figures S4** **,** **S5** show the variations of the number of upregulated and downregulated genes along with the different thresholds, respectively. For most cancer types, the significant enrichments of upregulated genes (tumor vs. normal) in the pathways of the GoF cluster were observed (**Figure 3A**, hypergeometric test, *p* < 0.05). The pathways whose members did not overlap with the DEG (upregulated or downregulated) are not shown in **Figures 3A, B**. Similarly, downregulated (tumor vs. normal) genes were significantly enriched in the pathways of the LoF cluster (**Figure 3B**, hypergeometric test, *p* < 0.05). The significant enrichments validated the aforementioned MoA hypothesis of GoF and LoF across pan-cancer. Identifying the MoA of cellular pathways was crucial to exploring the opportunities to therapeutically target pathways, because in principle, only pathways with activity (LoF, GoF, or SoF) could be directly targeted with molecules able to inhibit their dysfunctions.

### Characteristics of High Mutation and Target Enrichment Concentrate Seven Important Pathways Across Pan-Cancer

To detect the important KEGG pathways in each cancer, we proposed the following two questions: (1) whether significantly mutated genes (SMG) were enriched in the GoF pathways. The assumption proposed by Gonzalez implied that mutations in certain genes provided a selective advantage to tumor cells that followed by growing and proliferating faster (26). Tracing the signals left by the selection benefited to identify the gene driving tumorigenesis (27). Therefore, the enrichments of mutational drivers on GoF pathways suggested the pathway driving tumorigenesis. (2) Considering the pathway-targeted therapies for cancer, we asked the second question, whether drug targets (DT) were enriched in the GoF pathways. The enrichments of common DTs on GoF pathways indicated their potential of benefiting in cancer treatments.

The SMG list consisted of 585 driver genes collected from two pan-cancer studies (28). The list of 1,869 DTs was curated from DrugBank which currently consisted of ~10,000 drug entries (29, 30). The hypergeometric test was performed for three MoAs of pathways, and the statistically significant *p* values are as shown in **Figures 3A, B**. Using the threshold of *p* values above 0.05 (hypergeometric test), SMG were enriched in 47 pathways and DT were enriched in 15 pathways. GoF pathways contributed to 11 and 7, respectively. Intersecting the upregulated and downregulated significant pathways with DT-enriched pathways showed two and eight pathways, respectively. Intersecting the upregulated and downregulated significant pathways with SMG-enriched pathways showed six and five pathways, respectively (**Figure 3C**, left panel). There were 7 pathways that were enriched for both mutational and drug-targeted evidence. The enriched pathways are involved in five cancer-related diseases, a metabolic disease, and two signaling pathways (**Figure 3C**, right panel). The only one pathway in the GoF cluster which was both DT-enriched and SMG-enriched was “Type_II_Diabetes_Mellitus,” indicating that the metabolic syndrome increased cancer risk which is consistent with the perspectives in the previous studies (31, 32).

### Quantifying the Magnitude of the Pathways Across 20 Cancers and Within Specific Cancer

#### Twenty-Two Generalized, Important Pathways Across 20 Cancer Types

To quantify the magnitude of biological pathways across 20 cancer types, we defined a generality score which assigned the functional inclination of enrichment or depletion to pathways. The “generality score” quantifies the consistency of deregulated pathways across different cancer types and is a measure of the extent to which the levels of the pathway were perturbed by various cancer types. The higher the absolute value of the generality score, the larger the magnitude of a gene set or a pathway is. The sign of generality means the enrichments or depletions which indicated the positive or negative functional inclination in most cancers.

The generality scores for each pathway in the three clusters of three MoAs are shown in **Figure 3D**. The top 11 generally enriched and depleted pathways across various cancers are shown in **Table 2**. The most common enriched pathways were implicated in the fundamental biological processes maintaining the cell survival, cell cycle, DNA replication, and homologous recombination. In contrast, majority of common depleted pathways were the metabolic processes including tryptophan, propanoate, fatty acid, and tyrosine. The distribution of all generality scores across 178 KEGG pathways is shown in **Figure S6**.

**Table 2:** The top 11 generally enriched and depleted pathways across various cancers.

Depleted pathways	Generality score	Enriched pathways	Generality score
Fatty_Acid_Metabolism	-10.5	Basal_Transcription_Factors	10.5
Propanoate_Metabolism	-10.5	Bladder_Cancer	10.5
Tryptophan_Metabolism	-10.5	Cell_Cycle	10.5
Adipocytokine_Signaling_Pathway	-10.45	Pyrimidine_Metabolism	10.5
Beta_Alanine_Metabolism	-10.45	Dna_Replication	10.45
Butanoate_Metabolism	-10.45	Homologous_Recombination	10.45
Glycine_Serine_And_Threonine_Metabolism	-10.45	Nucleotide_Excision_Repair	10.45
Ppar_Signaling_Pathway	-10.45	P53_Signaling_Pathway	10.45
Primary_Bile_Acid_Biosynthesis	-10.45	Progesterone_Mediated_Oocyte_Maturation	10.45
Tyrosine_Metabolism	-10.45	Purine_Metabolism	10.45
Valine_Leucine_and_Isoleucine_Degradation	-10.45	Small_Cell_Lung_Cancer	10.45

To verify whether the generality could represent the features of the common pathways across 20 cancers, we counted the numbers of cancer types in which the NES was above 1 for the above 22 generalized pathways (**Table 2**). All the generalized, depleted pathways were observed to have NES of less than -1 in more than half of 20 cancer types. 9/11 of the depleted pathways (butanoate metabolism, valine leucine, and isoleucine degradation) were observed to have NESs of less than -1 in the third quartile (top 75%, 15 cancer types) of NES across 20 cancer types. As for the generalized, enriched pathways, they were all observed to have NESs exceeding 1 in more than half of 20 cancer types. 1/11 of the enriched pathways (basal transcription factors) was observed to have NESs of more than -1 in the third quartile (top 75%, 15 cancer types) of NESs across 20 cancer types. **Figure S7** shows the NESs of each pathway across 20 cancers.

According to the generality profiles in **Figure 3D**, we could reassign 4 pathways to the new MoA cluster more exactly (**Figure 3D**). In hierarchical clustering, we labeled O-glycan biosynthesis as the LoF cluster. However, the generality of 7.2 showed the proximity of cluster GoF. The “glyoxylate and dicarboxylate metabolism,” “alanine aspartate and glutamate metabolism,” and “selenoamino acid metabolism” were reassigned to the LoF cluster in spite of being possessed by the GoF cluster in the unsupervised clustering in the former.

Therefore, the generality we proposed was a novel measure to reflect the commonness of a gene set or a pathway among multiple cancer types. Generality benefits the development of pathway-targeted therapy in drug combination and prescription of herbal medicine.

#### Tumor-Specific, Important Pathways for Each Cancer

The pathway-targeted therapy required detections of not only the generalized or shared pathways but also tumor-specific pathways activated in specific cancer (33). Intratumor heterogeneity explained differences both in molecular phenotype and clinical outcomes (34). The follow-up data from TCGA showed KICH with the highest 5-year survival rate (up to 73%) and STAD with the lowest 5-year survival rate (merely 13%). The survival and matched transcriptomic data (RNA-seq) from TCGA enabled us to perform the pathway-level survival analysis to identify prognostic pathways for each of the 20 cancer types. The pathway-level survival analysis revealed the prognostic implication of pathways which reflect the molecular phenotype in specific tumor.

**Figure 4:**
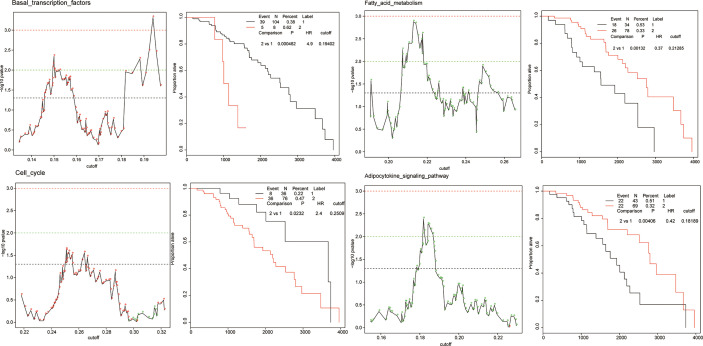
The examples of prognostic pathways identified in survival analysis in BRCA. Adverse prognostic pathways: basal transcription factors, cell cycle; favorable prognostic pathways: fatty acid metabolism, adipocytokine signaling pathway. The adverse and favorable pathways are defined as follows: 1) favorable prognostic pathways, for which a higher PA score of a pathway was correlated with a good/longer survival outcome, and 2) adverse prognostic pathways, for which a higher PA score of a pathway was correlated with a poor survival outcome.

To quantify the activation of each cellular pathway in specific patients, we defined a pathway activation (PA) score by single sample extension of GSEA (ssGSEA). ssGSEA measures the overexpression of a pathway in each patient by comparing the rank of the genes in the pathway with all other genes. The patient cohort of each cancer was stratified into two groups according to a given threshold of the PA score. To obtain the optimal cutoff, we iteratively applied a series of thresholds to a log-rank test, examining the most significant differences in survival outcomes between two groups. The PA score with the lowest log-rank *p* value was considered as the optimal cutoff. In total, 3,560 Kaplan–Meier (KM) plots corresponded to all 178 KEGG pathways across 20 cancer types. **Figure 4** shows the cellular pathways which were generalized enrichments or depletions in breast cancer according to the generality score.

The cellular pathway of a cancer was defined as a prognostic pathway with PA scores higher or lower than the optimal cutoff in an individual patient which yields a significant (*p* < 0.001, log-rank test) difference in overall survival. Totally, 319 significant prognostic pathways were defined in 20 cancers. The number of unique prognostic pathways was 152. We detected two types of prognostic marker pathways in terms of clinical outcomes: (1) favorable prognostic pathways, for which a higher PA score of a pathway was correlated with a good/longer survival outcome, and (2) adverse prognostic pathways, for which a higher PA score of a pathway was correlated with a poor survival outcome. The proportion of favorable and adverse prognostic pathways varied in different cancer types.

By uncovering tumor-specific and generalized prognostic pathways in pan-cancers, exploring the magnitude of the cellular pathways helps to accelerate the discovery of targetable pathways in cancers and facilitate generations of novel hypotheses.

### A Strategy for Computational A Strategy for Computational Drug Prescription in Cancers (CDP)

To connect therapeutic agents with pathways, we introduced the 1,309 perturbations (approved drugs, small-molecule compounds, and herbal medicines) obtained from the connectivity map (cMap build 02, https://clue.io/cmap) as a drug library. In each cancer type, we performed “cMap analysis” (35) to obtain the normalized enrichment scores (NESs) of all 1,309 perturbations, which suggests the possible treatment efficiency scores based on transcriptomic level. Across all the 20 cancer types, a matrix consisting of 1,309 × 20 was produced and defined as perturbation-related NES (PNS).

**Figure 5:**
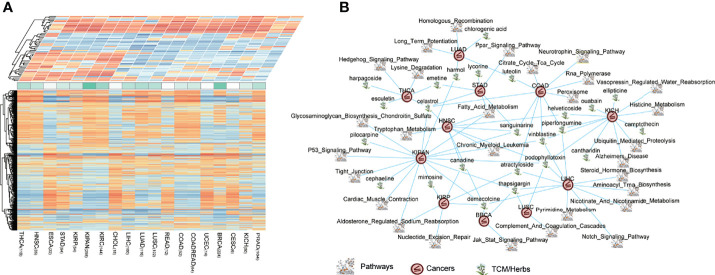
The connections of cancers–drugs–pathways. **(A)** The heterogeneous panels including perturbations (bottom panel, PNS matrix) and pathways (top panel, CNS matrix) associated with cancer types. A matrix consists of 1,309 drugs, and 20 cancers (1,309 × 20), representing the potential relationship of treatments, were produced and defined as perturbation-related NES (PNS). A matrix consists of 178 pathways, and 20 cancers (178 × 20), representing the dysregulation (enrichment or depletion) of each pathway in each tumor type, were defined as the cancer-related NES matrix (CNS). **(B)** The pan-cancer reversing network performed by Cytoscape, including 63 drug–pathway–cancer triplets.

According to the same cancer type, we combined CNS that we have defined before with PNS, to connect all possible pairwise perturbations and pathways. We constructed a landscape of heterogeneous panels including perturbations and pathways associated with cancer types (**Figure 5A**). The heterogeneous panels might provide evidence of possible effects of perturbations and pathways on specific cancer type. The basic hypothesis is that there are oncogenic and prognostic pathways in which the gene expression level is significantly upregulated in cancers, and the potentially efficacious drugs are those perturbations which possess the capability of reversing the upregulated pathway signatures. This theory is similar to that of cMap, in which using a rank-based pattern-matching strategy is based on a small list of genes as a query signature (35, 36).

To achieve the goal of prescription, i.e., screening the perturbations that are potentially useful for some cancer type on some pathway, we built a prescription score (PS) to measure the reversal potency of corresponding perturbations for a significant, prognostic pathway in some cancer type using the CNS and PNS matrix. The calculation was shown as follows: PS = absolute|CNS - PNS|. The construction of CNS and PNS and the calculation of PS consist of the strategy for computational drug prescription in cancers (CDP).

To extend the CDP strategy to application, we focused on traditional Chinese medicines (TCMs or herbs, herbal medicines). The first application of the CDP pipeline is on drug prescription for herbal medicines in traditional Chinese medicine. There were totally 120 herbal medicines in the cMap drug library. We identified prioritized herb–pathway–tumor triplets across pan-cancers and constructed a reversing network (**Table S2**, **Figure 5B**). The threshold to obtain the prioritized herb–pathway–tumor triplets consists of (1) a pathway that is significant, prognostic in survival analysis in at least one cancer type and (2) the absolute of the CNS score above 1.5 which means a pathway is enough upregulated or downregulated in the cancer. The network which is composed of prioritized cancer–drug–pathway triplets is illustrated in **Figure 5B**. The pan-cancer reversing network consisted of 63 drug–pathway–cancer triplets, involving 30 herbs and 42 significant, prognostic pathways across 13 cancer types.

Computational drug prescription in cancers (CDP) is a system-based approach to quantify the reversed effects of perturbations on tumors and predict the drugs showing high potency to reverse pathway activity in pan cancer.

## Discussion

In this study, we firstly constructed a profile of molecular dysregulation at the pathway level. Then we portrayed the mode of action (MoA) influenced by compounds and herbal components on pathways and identified oncogenic, prognostic pathways in which the gene expression level is significantly unregulated in cancers and adverse to good prognosis of patients. The MoA comprehensively represented characteristics of distinct pathways, implying the capacity of becoming drug targets. LoF and GoF are commonly changed in some cancers, and drugs targeting these pathways can be shared among these cancers, while drugs targeting SoFs should be carefully investigated before further researches, since one pathway in the SoF cluster represents different profiles based on gene expressions. By reversing the expression of the upregulated, prognostic pathways, we developed a strategy for *in silico* drug prescription on cellular pathways in cancers (CDP) to achieve the goal of drug prescription, such as reposition of the well-known drugs for new indications and discovery of herbal components indicating for tumors. It is noted that the predicted results could only provide an alternative unless an indication was validated *in vitro* and *in vivo*.

Applying CDP to TCM, we constructed the pan-cancer reversing network which consists of herb–pathway–tumor triplets. To give evidence of the application, we searched well-described relationships of herbs–cancers in the published literatures. With the corresponding pathways, the molecular mechanism of the herbs could be further explored.

Taking BRCA as example, the significantly prognostic pathway signature consisted of 13 cellular pathways, of which the most upregulated, dysregulated pathway was “Basal_Transcription_ Factors.” According to the PS value calculated by the CDP strategy, we focused on the top 3 herb medicines (lycorine, podophyllotoxin, cephaeline) that potentially reversed the pathway of “Basal_Transcription_Factors” (**Table 3**). The published evidence indicating the treatment on BRCA is shown as follows.

**Table 3:** The top PS value calculated by the CDP strategy.

Herbal medicines	PNS	CNS	Effects on cancers	PS	Prognostic pathways
Lycorine	-1.96614484	1.2588	Enrichment	3.224945	Basal_Transcription_Factors
Podophyllotoxin	-1.8677839	1.2588	Enrichment	3.126584	Basal_Transcription_Factors
Cephaeline	-1.486685	1.2588	Enrichment	2.745485	Basal_Transcription_Factors
Cephaeline	-1.31212566	1.0742	Enrichment	2.386326	Cytosolic_Dna_Sensing_Pathway
Emetine	-1.292820115	1.0742	Enrichment	2.36702	Cytosolic_Dna_Sensing_Pathway
Thapsigargin	2.0442182667	-1.4763	Depletion	-3.52052	Arachidonic_Acid_Metabolism
Esculetin	1.8627699667	-1.4763	Depletion	-3.33907	Arachidonic_Acid_Metabolism
Demecolcine	1.7208179	-1.5188	Depletion	-3.23962	Jak_Stat_Signaling_Pathway
Thapsigargin	1.6467795333	-1.5188	Depletion	-3.16558	Jak_Stat_Signaling_Pathway

Lycorine is a natural alkaloid extracted from the *Amaryllidaceae* genus. Ying et al. showed the suppression on cell growth, migration, and invasion both in multiple cell lines like MCF-7, T47D, MDA-MB-231, and 4T1 and in xenograft models (37). Besides the *in vitro* and *in vivo* studies, they also proposed that the mechanism of inhibition was that the blockage of Src/FAK (focal adhesion kinase), which is localized in nuclei, could play roles in regulating transcription factors (38). Similarly, Wang et al. also showed the findings about the inhibition of tumor growth and metastasis in breast cancer both *in vitro* and *in vivo* and investigated that suppressing STAT3 phosphorylation *via* upregulating SHP-1 expression was the cause of antitumor effects (39). Both the previous efforts provided the evidence that lycorine affected the regulations of transcriptional factors, which was represented as Basal_Transcription_Factors pathway in our CDP predictions.

Podophyllotoxin is an active component purified from *Podophyllum peltatum* (40). In terms of biological effects, podophyllotoxin is known to have immunosuppressive activity and antiviral effects against herpes, measles, influenza, and venereal warts (41). An *in vitro* study showed that podophyllotoxin presented the growth inhibitory activity for breast cancer cell lines and the inhibition extent up to 50% (42). Podophyllotoxin derivatives were investigated to inhibit the growth of both the breast cancer cell line MCF-7 and small-cell cancer cell line H69AR (43). The molecular mechanisms of podophyllotoxin in breast cancer were not investigated in current studies. However, in lung cancer, the anticancer effects of synthetic podophyllotoxin derivatives were verified *in vitro* and achieved by triggering mitotic arrest and ER stress (44).

Cephaeline was an analog of the natural product emetine, which was a kind of terpenoid tetrahydroisoquinoline alkaloids (45). Emetine is a protein synthesis/translation inhibitor that was evaluated in Phase II clinical studies as a potential chemotherapeutic agent for the treatment of solid tumors over 30 years ago (46). We did not find the literatures which reported the roles of cephaeline in BRCA, but Muhammad et al. indicated that cephaeline was cytotoxic to SK-MEL, KB, BT-549, and SK-OV-3 human cancer cells (45). Geir et al. proposed that cephaeline could be an inhibitor of tumors with necrosis, which was inferred from gene expression in aggressive endometrial cancers (47). In breast tumors, necrosis has been related to high-grade disease, increased tumor size, estrogen receptor negative status, high microvessel density, and macrophage infiltration (48). This evidence supports that our CDP strategy facilitates the drug prescription, especially for herbs, whose molecular mechanisms remain largely unknown.

There is a well-known computational method called cMap to connect diseases and small molecules. Using a list of differentially expressed genes (both upregulated and downregulated genes) in comparison of cases versus controls, cMap applies a computational method based on the Kolmogorov–Smirnov test to associated small molecules with the gene list, which reflected disease-related phenotypes. Our CDP has an advantage over cMap by taking biological pathways, especially prognostic pathways, into consideration, which approaches more to the nature of diseases.

## Conclusion

In summary, we developed a score system for *in silico* drug prescription on cellular pathways in cancers (CDP) by constructing a profile of molecular dysregulation at the pathway level. Then, we portrayed the three modes of action (MoA), which were influenced by compounds and herbal components on pathways including LoF, GoF, and SoF. Application of this system to herbal components derived from TCM or herbs revealed potential indications targeting cellular pathways in pan-cancers, deepening our understanding of the molecular mechanisms of herbs. In addition, we could also repurpose the well-known (Western) drugs into new indications, which not only saves time and cost in the development process but also avoids side effects. The computational pipeline complements to the traditional target-based approaches at the pathway level, connecting cancers with potential effective drugs.

## Materials and Methods

### Data Source

#### Transcriptomic Data

Paired tissue samples (tumor and normal) across 1,626 individual cohorts were obtained from TCGA using the R package of “TCGA2STAT.” The pan-cancer-normalized gene-level RNA-Seq data for the TCGA cohorts were arranged, as shown in **Table 1**. With the transcriptomic data, the clinical data for all the TCGA cohorts were also obtained for survival analysis.

#### Agent-Perturbed, Transcriptomic Data

The CMap database (build 02): four types of human cancer cell lines (MCF7, PC3, HL60, SKMEL5) were treated with 1,309 distinct perturbations including US Food and Drug Administration (FDA)-approved drugs and uncharacterized bioactive compounds (called perturbagens by the authors of cMap, here simply referred to as drugs), for a total of 6,097 treatments. Gene (total RNA) expressions from the 6,097 “instances” (an instance is a cell line treated with a drug at a dosage, and its non-treated control) were recorded in two batches of microarrays: 671 HG-U133A (Affymetrix) chips (on 407 drugs) and 5,426 HT-HG-U133A chips (for a total of 6,097 chips on 1,309 drugs). Raw data were downloaded from the CMap website (http://www.broadinstitute.org/cmap/).

#### Cellular Pathways

Cellular pathways are a collection of manually drawn KEGG pathway maps representing experimental knowledge on metabolism and various other functions of the cell and the organism. Each pathway map contains a network of molecular interactions and reactions and is designed to link genes in the genome to gene products (mostly proteins) in the pathway.

### Construction of Activity Profiles of Pathways

We employed Gene Set Enrichment Analysis (GSEA) to connect cancers to biological pathways from Kyoto Encyclopedia of Genes and Genomes (KEGG). We took the 186 KEGG pathways as the representatives of the general physiological processes. The pathway list was narrowed down into 178 pathways since those members of 8 pathways lacked gene expression levels in the paired pan-cancer cohorts. The NES (normalized enrichment scores) for 178 pathways were presented in a 178 × 20 table (**Table S1**), representing the dysregulation (enrichment or depletion) score of each pathway in each tumor type. This cancer-related NES matrix was referred to as **CNS**. On the basis of 1,000 trials, the empirical *p* values for the observed enrichment of 178 KEGG biological pathways were obtained. The false discovery rate (FDR) correction was used to deal with the accumulation of type I errors (false positives) in multiple comparisons.

### Calculation of Generality Score

Generality is a measure that quantifies the deregulated commonness of a gene set or a pathway among multiple cancer types. Let *n* be the total number of cancer types in a study (20) and *m* the number of gene sets or pathways (178). For each pathway *i* in a collection of pathways, both *a* and *b* statistics are respectively computed corresponding to enriched pathways and depleted pathways, giving *a_i_* and *b_i_*. Actually, the generality score could be calculated for any gene sets or pathways. For simplicity and easy understanding, we only referred to pathways.

All cancer types in an ascending order were sorted by the normalized enrichment score of the gene set *i*, giving *r_ik_* the rank of gene set *i* in the *k^th^* cancer type, where *k* = 1, 2,…, *n*. A rank matrix (*m* rows × *n* columns) of the position (1… *n*) of each pathway in certain cancer was constructed. The ranks *r_ik_* of *n* cancer types for *m* pathways in the current analysis are contained in the **Supplementary File**.

The *a* and *b* statistics were weighed using the proportion of significant enrichments and depletions in *n* cancer types of pathways *i*. Significant enrichments of pathway *i* require that the NES is positive and significance *p* is less than 0.05 in certain cancer type *k*. Significant depletions of pathway *i* require that the NES is negative and significance *p* is less than 0.05 in certain cancer type *k*. The number of cancer types for pathway *i* was counted according to the significant enrichments and deletions, giving *w_1_* and *w_2_*.

The following two statistics corresponding to the enrichments and depletions of pathway *i* across *n* cancer types are computed:


$$a_{i}=\frac{w_{1}}{w_{1}+w_{2}}\sum_{k=1}^{n}\left(\frac{r_{ik}}{n}\times 1\right)$$

$$bi=\frac{w_{2}}{w_{1}+w_{2}}\sum_{k=1}^{N}\left(\left(1-\frac{\left(r_{ik}-1\right)}{n}\right)\times (-1)\right)$$

$$generality\;score\;S_{i}=\{\begin{array}{c} a_{i}(|a_{i}|>|b_{i}|)\\ b_{i}(|a_{i}|<|b_{i}|)\\ 0(|a_{i}|=|b_{i}|) \end{array}$$

Set *S_i_* = *a_i_*, if |*a_i_|* > |*b_i_* |, set *S_i_* = *b_i_* if |*a_i_* | < |*b_i_* |. The generality score is set to zero where *a_i_* and *b_i_* have the same value, which means that pathway *i* is not a generally deregulated pathway across these *n* cancer types.

### Survival Analysis

All the patients along with the survival information were included in the Kaplan–Meier (KM) survival analysis. The patients who lacked survival information were excluded from survival analysis. The KM curves show the differences in overall survival for patients stratified by an ssGSEA score (49) of a certain pathway for each cancer type (see details in **Supplementary Files**). The KM curve separation was assessed by the log-rank test. The pathways which achieved p < 0.001 were considered as prognostic pathways. For each cancer and each pathway, the patient cohort was stratified into two groups with the higher and lower enrichments (ssGSEA), and an optimal split was selected by tracing each ssGSEA score to find the smallest *p* of the log-rank test. If the activations of a pathway were measured for each patient, his or her survival time and status (alive or dead) will be used to distinguish the prognostic implications.

### Clustering Analysis

Pathway classification was identified using hierarchical clustering 178 pathways with the normalized enrichment score (NES) across all tumor types. The hierarchical clustering used the Spearman correlation metric and average linkage. The modes of action of pathways were identified using agglomerative hierarchical clustering of pathways with an SD > 1.0 on the log2 scale across all pathways. Consensus was commutated using 500 samplings of the pathways, 80% of the pathways per sample, agglomerative hierarchical clustering, Euclidean distance metric, and average linkage.

### Differentially Expressed Analysis

To test for the differential expression across two groups (tumor and normal), we used the R package samr on gene expression profiles (FPKM value) from TCGA RNA-seq datasets. The p values were adjusted for multiple testing based on the false discovery rate (FDR). For comparison of two sample groups, we applied SAM (significance analysis of microarrays) which consists of a modification of the standard t-test by the inclusion of an extra variable s_0_ called variance).

## Data Availability Statement

The original contributions presented in the study are included in the article/**Supplementary Material**. Further inquiries can be directed to the corresponding authors.

## Author Contributions

ZZ, YR and YL designed the work. KeZ, QX, RR,YP collected the data, FM, KZ, KenZ, CY, and YZ analyzed the data, YS, FM and ZZ interpreted results and wrote the manuscript, ZZ, YL, and HG reviewed the manuscript. FM participated in the preparation of the figures and tables. All authors contributed to the article and approved the submitted version.

## Funding

This work was supported by grants from the National Key R&D Program of China(No. 2017YFF0106006) and the National Natural Science Foundation of China (No. 82002647).

## Conflict of Interest

Authors FM, YP, KZ, QX, RR, YZ, YS, YL, HG, and YR were employed by CapitalBio Technology.

The remaining authors declare that the research was conducted in the absence of any commercial or financial relationships that could be construed as a potential conflict of interest.

## Publisher’s Note

All claims expressed in this article are solely those of the authors and do not necessarily represent those of their affiliated organizations, or those of the publisher, the editors and the reviewers. Any product that may be evaluated in this article, or claim that may be made by its manufacturer, is not guaranteed or endorsed by the publisher.
