# Supplementary figures and images for: Prognostic Pathways Guide Drug Indications in Pan-Cancers
Source: Front Oncol. 2022 Mar 14;12:849552. doi: 10.3389/fonc.2022.849552 (PMC8964428; doi:10.3389/fonc.2022.849552)

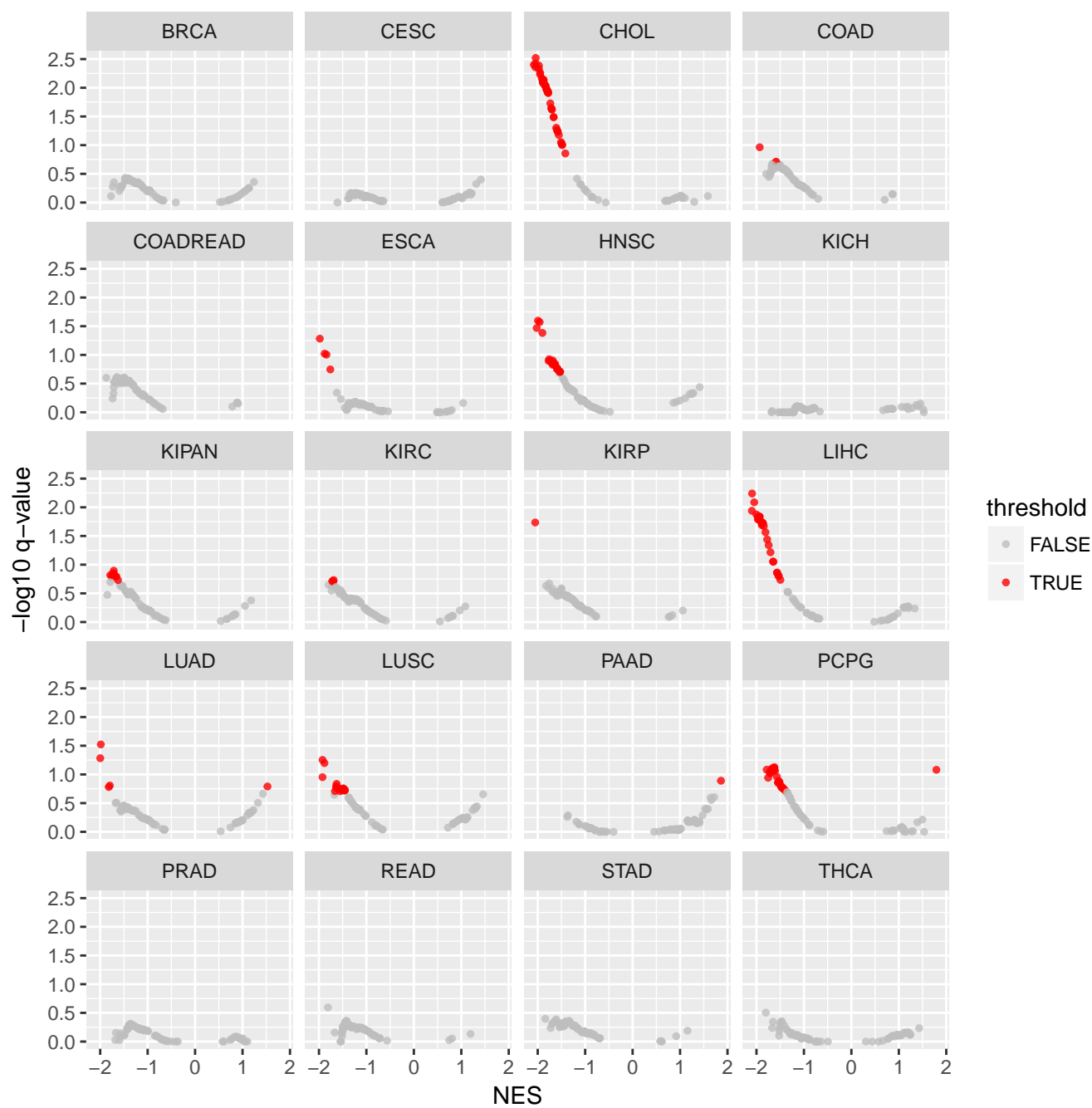

Supplement: Supplementary file 1 [file Image_1.pdf]

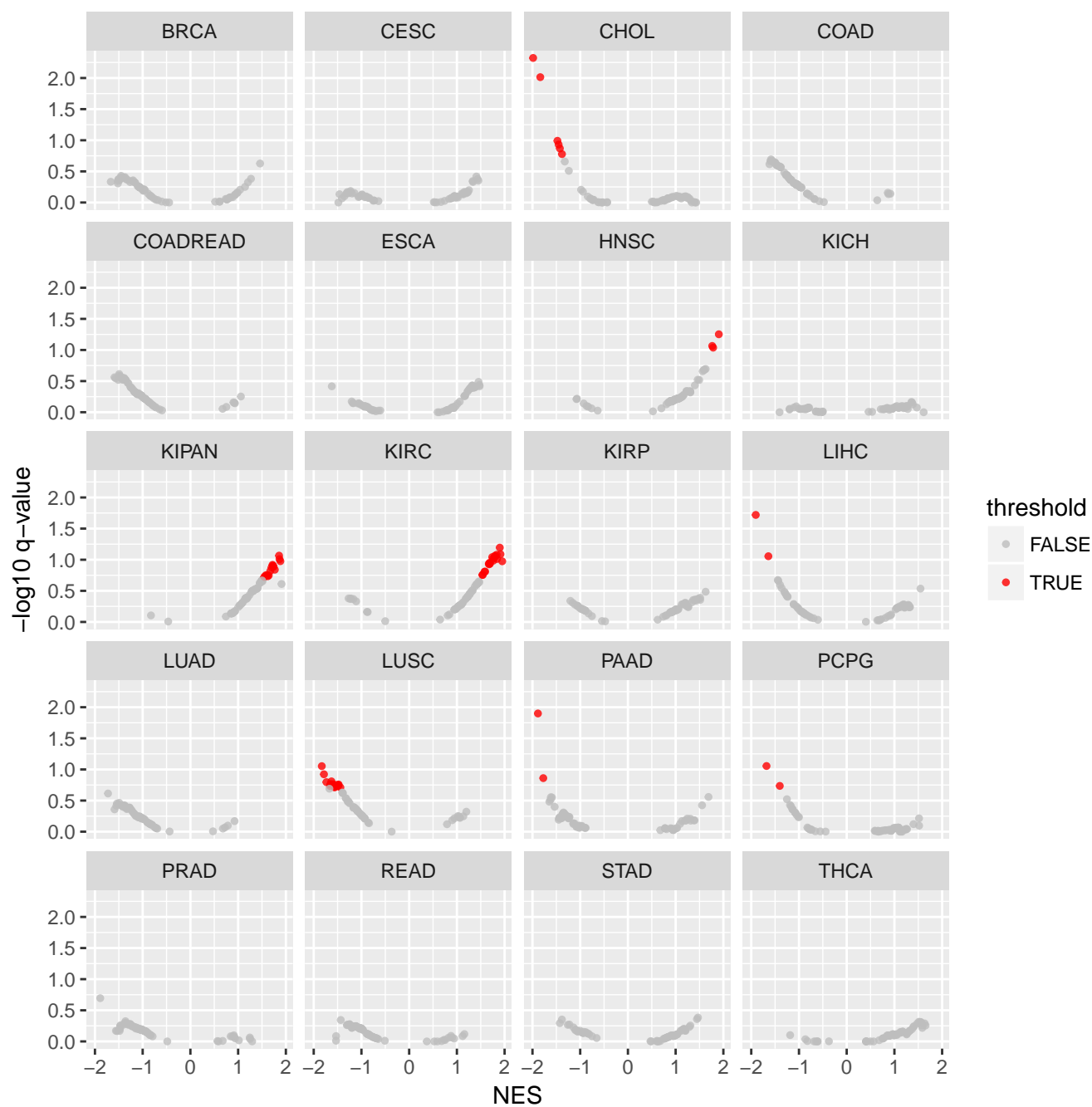

Supplement: Supplementary file 2 [file Image_2.pdf]

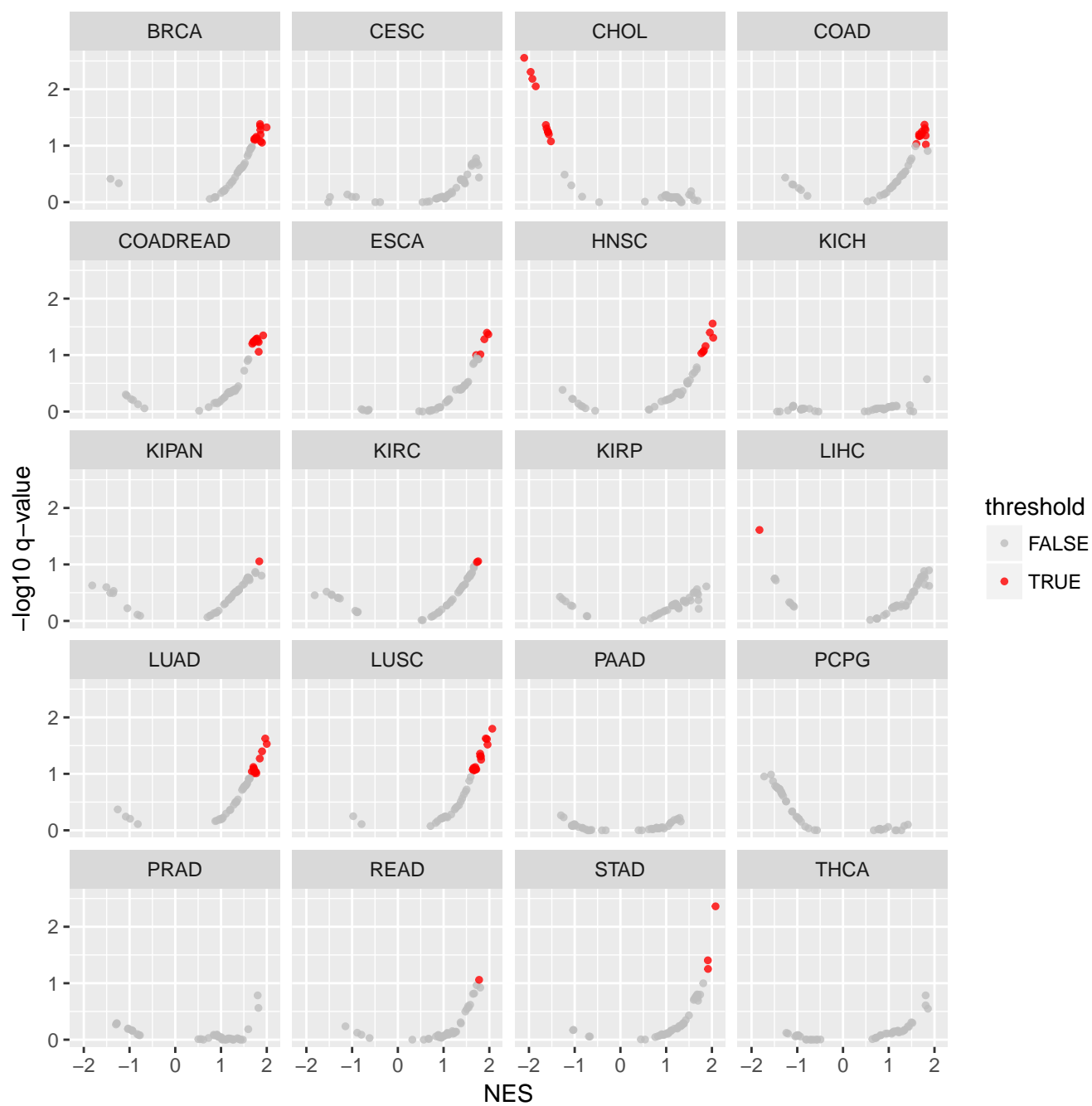

Supplement: Supplementary file 3 [file Image_3.pdf]

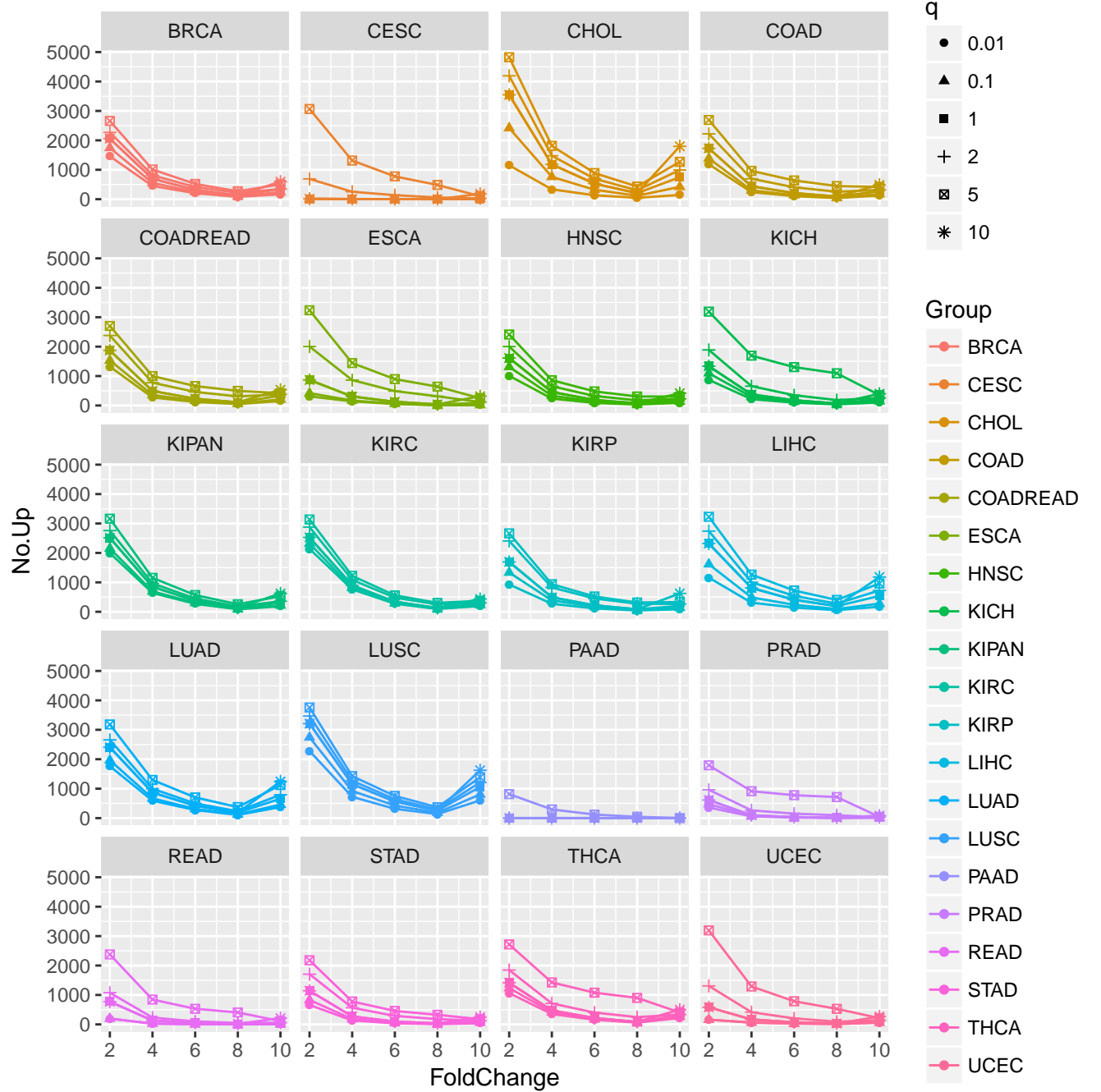

Supplement: Supplementary file 4 [file Image_4.pdf]

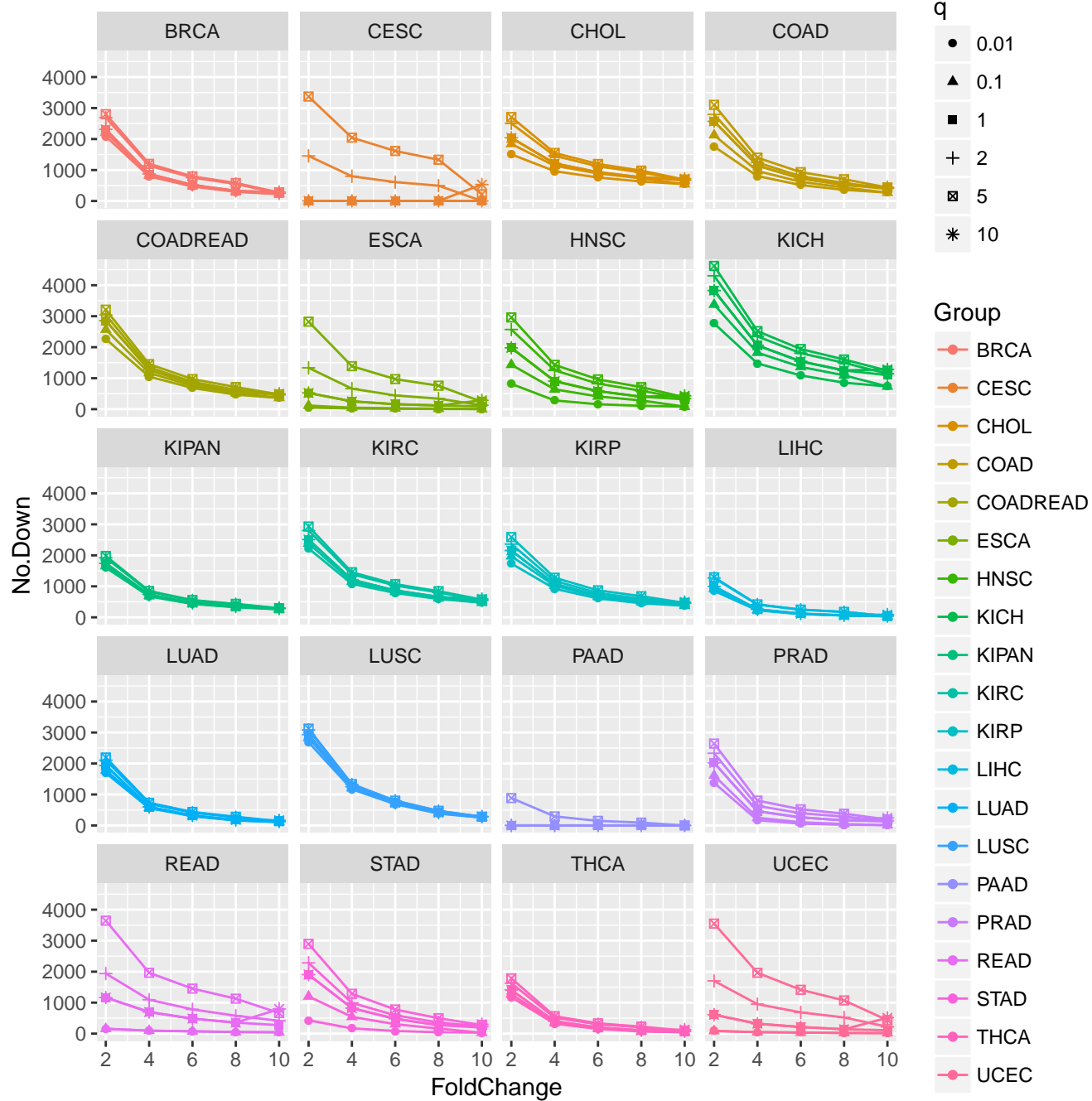

Supplement: Supplementary file 5 [file Image_5.pdf]

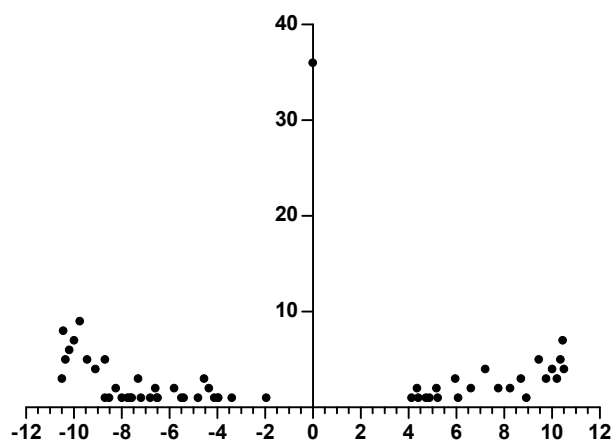

Supplement: Supplementary file 6 [file Image_6.pdf]

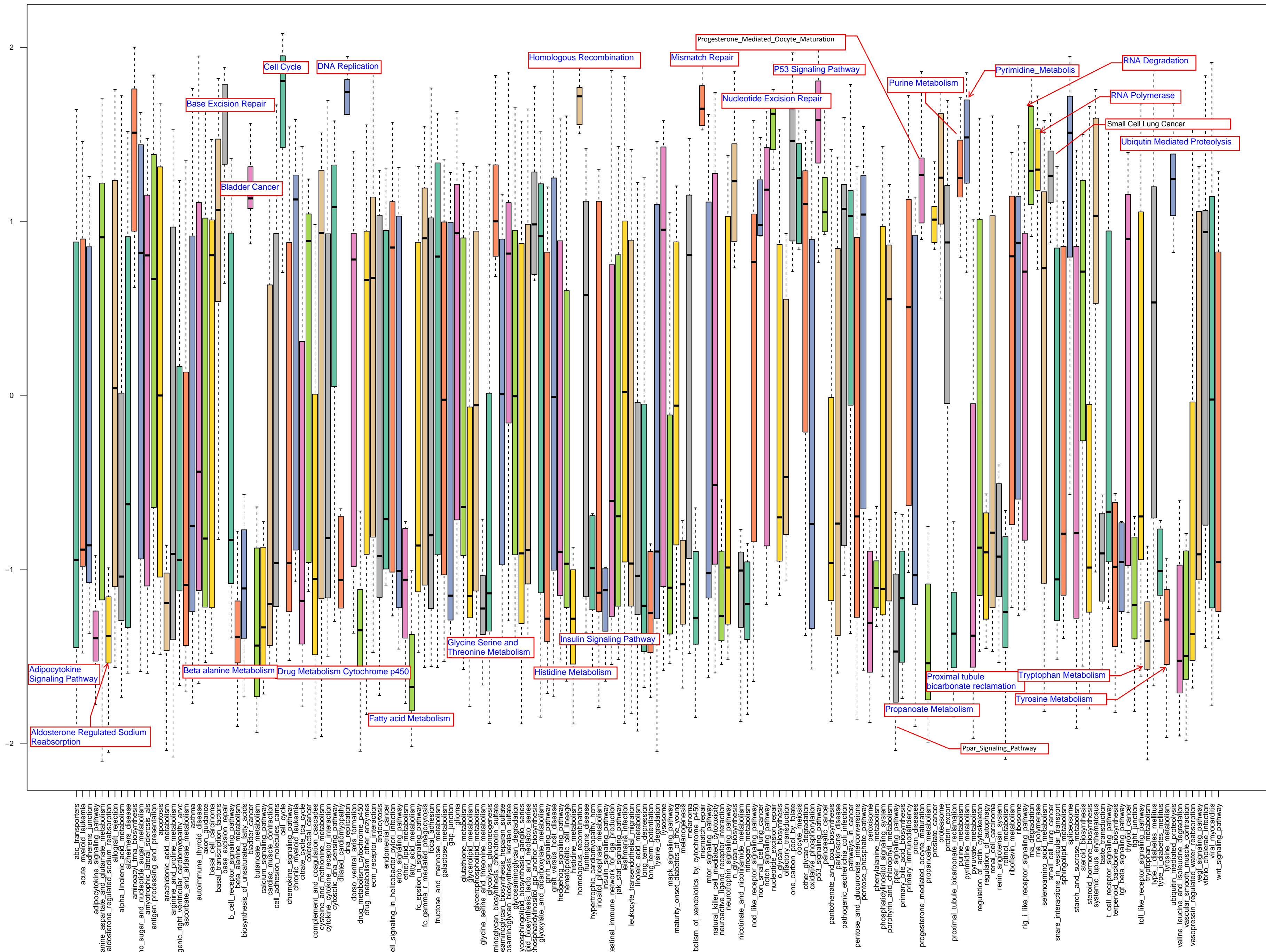

Supplement: Supplementary file 7 [file Image_7.pdf]
